# Promoting Co-Crystallization in Poly(butylene succinate) and Poly(butylene fumarate) Blends via End-Group Functionalization

**DOI:** 10.3390/molecules27207086

**Published:** 2022-10-20

**Authors:** Xue-Wei Wei, Cong Chen, Tian-Yu Wu, Li-Hai Cai, Hai-Mu Ye

**Affiliations:** 1 Department of Materials Science and Engineering, College of New Energy and Materials, China University of Petroleum, Beijing 102249, China; 2 Institute of Systems Engineering, AMS, Beijing 102300, China

**Keywords:** co-crystallization, polymer blend, hydrogen bond, compatibility

## Abstract

Co-crystallization plays a crucial role in the integration and regulation of thermal and mechanical properties in polymer blends, but the poor compatibility of the components in the crystal phase has always been a major obstacle to co-crystallization, which puts forward stricter requests for linkage and interaction between different entities. On the basis of the hydrogen-bonding interaction that can promote chain stacking and thus improve miscibility, we propose that crystalline/crystalline blends of 2-ureido-4[1H]-pyrimidinone (UPy)-functionalized poly(butylene succinate) and poly(butylene fumarate) (PBS-UPy/PBF-UPy) where UPy groups with quadruple hydrogen-bonding interaction are employed to connect different chain ends, could inhibit phase separation and improve co-crystallization. PBS-UPy/PBF-UPy blends exhibit complex component-dependent and cooling-rate-dependent co-crystallization behavior. A high level of co-crystallization occurs in the range of PBS-UPy-rich fractions, and the proportion could approach over 98% under optimized conditions with the aid of UPy quadruple hydrogen bonds interaction. This work enriches the understanding of co-crystallization in crystalline/crystalline polymer blends and provides more possibility for the design of structures and properties of polymer materials.

## 1. Introduction

Blends, mixtures of polymers, are a common strategy for combining and modulating thermal and mechanical properties without the need for complex synthetic procedures [1]. However, unlike covalently-bonded copolymers, the possible non-covalent interactions existing in blends are usually not strong enough to overcome phase separation between different components, especially in crystalline/crystalline polymer blends. The phase separation could be enhanced by crystallization, making the non-selective adaptation in the crystalline phase more difficult. Consequently, co-crystallization behavior in crystalline/crystalline blends usually occurs only under special requirements.

Co-crystallization behavior is an optimal crystallization state without significant degradation of crystallization properties and it has received fairly extensive attention. Most of the co-crystallization behaviors in polymer blends that have been found so far are based on components with similar molecular and crystal structures [2,3,4]. However, poor miscibility or incomplete co-crystallization is still inevitable, even though the two crystalline components possess similar structures [5,6], which places a higher demand on us to enhance the interaction and connection between different kinds of polymer entities.

Supramolecular interactions are an important means to facilitate the linkage of two components [7], and thus improve the miscibility in polymer blends. The most common supramolecular interactions include electrostatic and metal ion coordination [8,9], dipole-dipole interaction [10], and hydrogen bonds [11,12]. Among them, hydrogen-bonding interactions can promote compact chain stacking and thus improve miscibility. Even though the singlet inter-segmental hydrogen-bonds formed between N–H and C=O groups is weak, they can promote co-crystallization behavior in the blends of polyamide 6 (PA6) and polyamide 410 (PA410) when the fraction of PA6 in blend is less than 50% [13]. Therefore, it is likely that if multiple inter-segmental hydrogen-bonds were incorporated between different entities in polymer blends, co-crystallization would be promoted. The molecule of 2-ureido-4[1H]-pyrimidinone (UPy) is a widely employed unit to construct a strong hydrogen bond, and the UPy-UPy quadruple hydrogen bonds can display strength close to a typical covalent bond [14]. Thus, various polymers linked with UPy have been prepared and studied, and exhibit novel properties in comparison with the corresponding unmodified polymers. The blend of UPy end-group functionalized poly(*l*-lactide) (PLLA-UPy) and poly(*d*-lactic acid) (PDLA-UPy) exhibits a marked enhancement of stereocomplex crystallization (a unique co-crystallization method) and the suppressed crystallization ability of homo-PLA, because the dimerization of UPy groups randomly connects the PLLA-UPy and PDLA-UPy chains, leading to a dramatic increase in compatibility and miscibility between different components [15,16]. Meanwhile, UPy has been considered for use as a linker for the supramolecular design of polymers, including PLA [17], poly(ethylene glycol) (PEG) [18], poly(ε-caprolactone) (PCL) [19], and poly(butylene succinate) [20], which could supply the foundation for manipulating co-crystallization behavior in crystalline/crystalline blends.

Our previous work has demonstrated that the isomorphism of PBS and poly(butylene fumarate) (PBF) blends occurs in the range of PBS-rich fractions, and the fraction of co-crystals close to 75% can be achieved under the optimized condition with the aid of a weak hydrogen-bonding interaction [21]. Therefore, it is proposed that the co-crystallization degree in the PBS/PBF blend would be effectively increased once a much stronger interaction was introduced between these two entities, for example, multiple hydrogen bonds. High levels of co-crystallization ability are conducive to tuning the thermal and mechanical properties of crystalline/crystalline polymer blends with non-covalent linkages. In addition, the crystallization behavior of the supramolecular system constructed by different crystalline polymers has been less investigated. New and novel crystallization of such sophisticated systems and the relevant mechanisms remain to be elucidated.

Therefore, in this study, we synthesize UPy end-group functionalized PBS and PBF with similar molecular weights and systematically investigate the structure, thermal properties, and crystallization behavior of their blends. It is demonstrated that the strong hydrogen-bonding interaction provided by UPy end-groups could effectively promote the co-crystallization in the blend, which exhibits specific component-rate-dependent and cooling-rate-dependent crystallization behavior. This study helps facilitate the realization of the regulation of co-crystallization in crystalline/crystalline polymer blends.

## 2. Results and Discussion

### 2.1. Chain Structure of Polyesters

**Figure 1:**
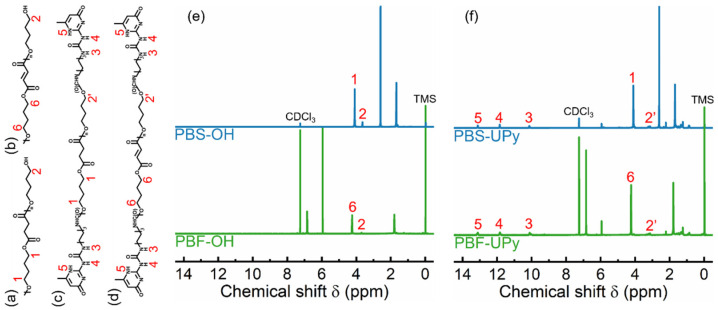
Chemical structures of PBS-OH (**a**), PBF-OH (**b**), PBS-UPy (**c**), and PBF-UPy (**d**), and their ^1^H NMR spectrum (**e**,**f**).

The ^1^H NMR spectra of various PBS and PBF samples are shown in Figure 1. The characteristic peak “1” at 4.11 ppm and peak “6” at 4.24 ppm are assigned to the methylene protons of butylene in PBS-OH and PBF-OH repeating units, respectively. Peak “2”, at around 3.6–3.7 ppm, belongs to the protons in terminal methylene groups [22]. While hydrogen bonding results in a downfield shift of the NMR signal of the proton [23,24], the UPy-terminated products show a movement of the peak to the slightly lower values of the chemical shift observed for the products, denoted by “2′”.

The weak peaks located at 10.1 ppm (peak “3”), 11.8 ppm (peak “4”), and 13.1 ppm (peak “5”) are attributed to the different protons in terminal UPy units [25]. The integrated areas of the characteristic peaks are provided in Table S1 of the supplementary information. Thus, the *M*_n_s of various PBS can be calculated by the following equations:

(1) $$M_{n}\left(\mathrm{PBS}-\mathrm{OH}\right)=90+\frac{I_{\mathrm{peak}\;1}}{I_{\mathrm{peak}\;2}}\times 172$$

(2) $$M_{n}\left(\mathrm{PBF}-\mathrm{OH}\right)=90+\frac{I_{\mathrm{peak}\;6}}{I_{\mathrm{peak}\;2}}\times 170$$

(3) $$M_{n}\left(\mathrm{PBS}-\mathrm{UPy}\right)=294*2+88+\frac{I_{peak\;1}}{2I_{\mathrm{peak}\;5}}\times 172$$

(4) $$M_{n}\left(\mathrm{PBF}-\mathrm{UPy}\right)=294*2+88+\frac{I_{\mathrm{peak}\;6}}{2I_{\mathrm{peak}\;5}}\times 170$$

where “*I*” represents the integral areas. The values of 172 and 170 are the molecular weights of repeating units in PBS and PBF, and the values of 90, 292, and 88 are the molecular weights of end groups of different PBS and PBF, respectively.

The *M*_n_s of PBS-OH and PBF-OH are respectively calculated out as 3070 g/mol and 2970, showing similar molecular weights. After termination by UPy units, the molecular weights of the PBS and PBF samples increase. The molecular weights of PBS-UPy and PBF-UPy are respectively determined as 3860 g/mol and 3790 g/mol, about 800 g/mol higher than the corresponding PBS-OH and PBF-OH. Considering the possible loss of low molecular weight fraction of the final product during the purification process, it is reasonable to consider that the chain ends of PBS-OH and PBF-OH are mostly substituted by UPy units in PBS-UPy and PBF-UPy.

**Figure 2:**
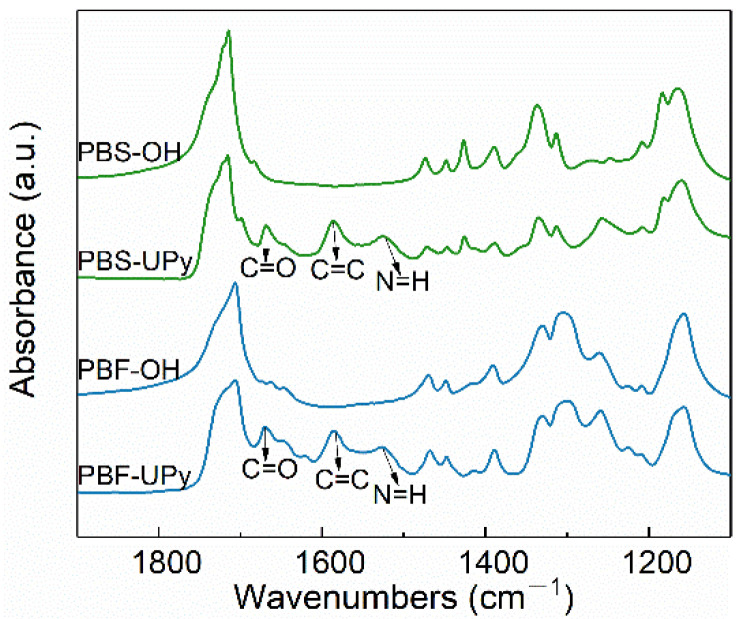
FTIR spectra of PBS-OH, PBF-OH, PBS-UPy, and PBF-UPy were collected at room temperature in the wavenumbers range from 1900 to 1100 cm^−1^.

The incorporation of UPy units was further confirmed by FTIR spectra. As shown in Figure 2, the additional absorption bands at 1668 cm^−1^ (C=O stretching vibration), 1587 cm^−1^ (C=C stretching vibration), and 1525 cm^−1^ (N–H bending vibrations) of PBS-UPy and PBF-UPy, in comparison with the hydrogen-terminated samples, were confirmed as being associated with the UPy units [26,27]. The FTIR and ^1^H NMR results corroborate each other, demonstrating the successful synthesis of PBS-UPy and PBF-UPy.

### 2.2. Thermal Properties of PBS-UPy/PBF-UPy Blends

**Figure 3:**
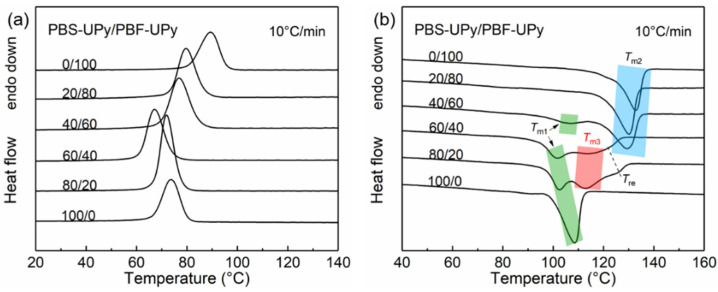
DSC curves of PBS-UPy/PBF-UPy blends during the melt-cooling (**a**) and the subsequent heating (**b**) scans at a rate of 10 °C/min.

To detect the thermal properties of PBS-UPy/PBF-UPy blends, DSC measurements were performed. Figure 3 shows the DSC curves with a constant ramping rate of 10 °C/min. During the melt-cooling process, each PBS-UPy/PBF-UPy blend exhibits a single crystallization temperature (*T*_c_), which is lower than the corresponding PBS-OH/PBF-OH blend with close molecular weights [21]. The reason could be attributed to the end-group confinement of UPy as commonly observed in many polymers [19]. All blend samples exhibited only one apparent exothermic peak even during the slow cooling process (Figure S1A,B), plausibly indicating there is no apparent occurrence of phase separation between different entities. Meanwhile, *T*_c_ and crystallization enthalpy (Δ*H*_c_) do not change linearly with the increase of the content of PBS-UPy (Figure S2). Especially the PBS-UPy/PBF-UPy-80/20 displays a sharp crystallization peak, and obviously higher enthalpy (Figure 3a). Such phenomena might be attributed to the presence of a strong intermolecular interaction between UPy units that promotes the co-crystallization behavior [16] to a certain extent, rather than merely being present as end-group confinement.

The subsequent heating curves with broad melting peaks, which result from the overlapping signals of different types of crystals, are shown in Figure 3b, [28]. Based on the melting process of co-crystal in PBS/PBF blends [21], it is easy to distinguish that *T*_m1_ and *T*_m2_ correspond to melting of PBS and PBF crystals, respectively, and that the melting peak at the lower temperature of PBS-UPy/PBF-UPy-40/60 is attributed to the melting of PBS crystals, which will be demonstrated based on crystal structure confirmation in the following section. *T*_m3_s at ~112.3 °C and ~111.7 °C are attributed to the co-crystals in PBS-UPy/PBF-UPy-60/40 and PBS-UPy/PBF-UPy-80/20 blends, respectively. In addition, a new melting peak (*T*_re_) appeared between *T*_m2_ and *T*_m3_, which should be the melting endothermic of the melt-recrystallized crystals from co-crystals [29]. The fitting method is presented in the supplementary information (Figure S3). When the heating rate is increased fast enough (e.g., 40 °C/min), the re-organization of crystals during heating could be completely inhibited, which eventually leads to the disappearance of the peak of *T*_re_ (Figure S4).

**Figure 4:**
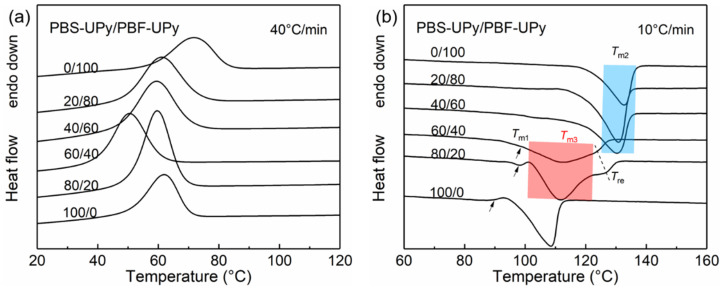
DSC curves of PBS-UPy/PBF-UPy blends during melt-cooling at a rate of 40 °C/min (**a**), and the subsequent heating scans at a rate of 10 °C/min (**b**).

To further determine the effect of UPy end groups on the co-crystallization between PBS-UPy and PBF-UPy, DSC measurements of melt-cooled samples at a faster rate of 40 °C/min were performed (Figure 4) and the thermal data are tabulated in Table 1. Compared with Figure 3 and Figure S1, it is clear that the formation of co-crystals in PBS-UPy/PBF-UPy blends is both cooling-rate- and composition-dependent. When the cooling rate is speeded up to 40 °C/min, the co-crystallization behavior in PBS-UPy/PBF-UPy blends is significantly promoted, with a fraction of the newly-appeared co-crystal close to 98% and 89%, respectively, in the blend of PBS-UPy/PBF-UPy-80/20 and PBS-UPy/PBF-UPy-60/40. As PBS-type crystal shows a higher accommodation ability for PBF than that of PBF-type crystal for PBS [21], PBS-UPy/PBF-UPy-80/20 exhibits a higher Δ*H*_m_ (45.5 J/g) among several sets of samples. The small endothermal peaks indicated by black arrows in Figure 4b might correspond to the melting process of imperfect crystals formed during the rapid cooling [30].

Here, the formation of co-crystal fraction (~98%) in PBS-UPy/PBF-UPy blend is remarkably promoted in comparison with PBS/PBF blend (~75%) [21]. Therefore, the complementary hydrogen-bonding interaction provided by UPy end groups should play a key role. Though the large stacks of UPy induced by hydrogen-bonds could suppress the crystallization, they enable the linkages between PBS chains and PBF chains, and thus help weaken the phase separation, which benefits the formation of co-crystals.

### 2.3. FTIR Investigation

**Figure 5:**
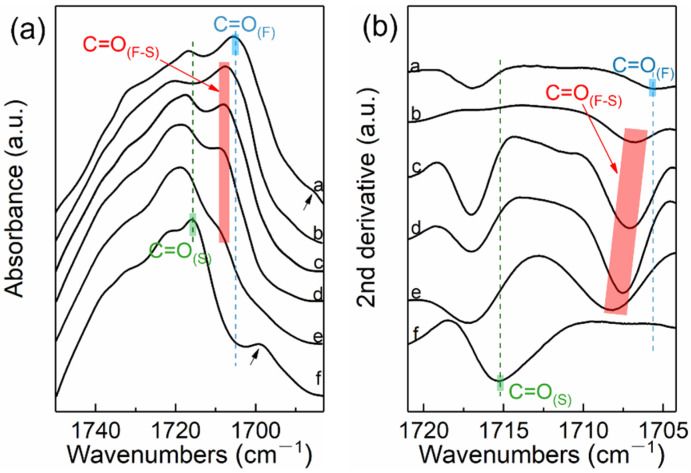
FTIR spectra of PBS-UPy, PBF-UPy and their blends in the wavenumber range of 1750–1685 cm^–1^ (**a**) and their second derivative spectra (**b**). Spectra a–f correspond to PBF-UPy, PBS-UPy/PBF-UPy-20/80, PBS-UPy/PBF-UPy-40/60, PBS-UPy/PBF-UPy-60/40, PBS-UPy/PBF-UPy-80/20, and PBS-UPy, respectively.

Figure 5 shows the FTIR spectra of PBS-UPy/PBF-UPy blends in the range of 1750–1685 cm^–1^ and their second derivative spectra. The absorption band at 1715 cm^–1^ is assigned to the C=O stretching vibration of the PBS crystalline phase (denoted by C=O_(S)_), [31] and the C=O vibration of the PBF crystalline phase at 1705 cm^–1^ (denoted by C=O_(F)_) [32]. However, the FTIR absorption of crystalline C=O stretching vibration in PBS-UPy/PBF-UPy blend crystalline phase is not only a simple superposition of C=O_(S)_ and C=O_(F)_, but includes a new, more independent absorption band, at around 1708 cm^–1^, which could be reasonably attributed to the interaction between the fumarate structure and the succinate structure (C=O_(F–S)_) [33,34]. The characteristic bands indicated by the black arrows at low wavenumbers correspond to the hydrogen-bonded C=O formed by PBS-UPy and PBF-UPy with UPy group, respectively [35]. Because there is a lot of overlap between the amorphous C=O vibration band of PBF and the C=O vibration band of PBS crystalline phase, in order to effectively identify the spectral difference, the second derivative spectra in the range of 1720–1705 cm^–1^ is employed (Figure 5b). All four PBS-UPy/PBF-UPy blends display an obvious C=O_(F–S)_ band, confirming that the two components of PBS-UPy and PBF-UPy are accommodated simultaneously in the crystal lattices of co-crystal. Furthermore, the gradually shifting behavior of C=O_(F-S)_ group could demonstrate the isomorphic behavior in the blends.

In addition, co-crystallization characteristics for PBS-UPy/PBF-UPy-80/20 blends can be revealed in detail by in situ FTIR results (Figure S5), in which the intensities of characteristic peaks correspondingly ascribed to the crystalline phase of PBS-UPy at 1425 cm^–1^ and PBF-UPy at 970 cm^–1^ changed uniformly and disappeared simultaneously when the temperature was raised to 124 °C. It is suggested that both PBS and PBF chains are involved in co-crystals and their proportions released during the melting process are almost constant, which is a piece of important evidence for isomorphism.

**Figure 6:**
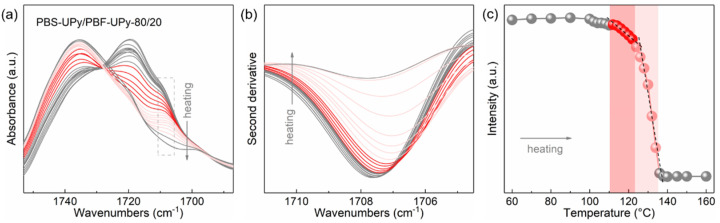
In situ FTIR spectra of blend PBS-UPy/PBF-UPy-80/20 (**a**) and their second derivatives (**b**) collected upon heating process from 60 to 160 °C after being melt-cooled at a rate of 40 °C/min, and the trend of characteristic peak intensity as a function of temperature (**c**).

Figure 6a shows the temperature-resolved C=O stretching vibration bands of PBS-UPy/PBF-UPy-80/20 during the step-heating process from 60 to 160 °C, which was melt-cooled at a rate of 40 °C/min in advance. To obtain more information on the evolution of C=O_(F–S)_ in the original spectra, the spectral resolution is enhanced by taking second-order derivative to magnify the difference (Figure 6b). For Lorentz or Gaussian–Lorentz peaks, the intensity of the second-order derivative is proportional to the intensity of the corresponding original peak [36], so the behavior of the co-crystal during melting can be traced by comparing the change in intensity of the second-order derivative. The C=O_(F–S)_ band remains almost stable until the temperature is raised to 100 °C. As the temperature is increased to 108 °C, its intensity decreases slightly, which is due to the melting of a small amount of less-stable crystals. Further, the co-crystals start to melt with the increasing temperature, and the C=O_(F-S)_ group intensity decreases nearly linearly, which is important evidence of the indiscriminate incorporation of the two components into the unit cell (marked in red in Figure 6c). We further compared the characteristic band attributed to the C=C stretching located at 1587 cm^−1^, which is sensitive to the UPy-UPy hydrogen-bonding interaction (Figure S6), and found that the intensity of this characteristic band is almost constant during the melting of the co-crystals. Therefore, it is suggested that the specific hydrogen-bonding interaction based on UPy groups in the PBS-UPy/PBF-UPy blend could be maintained during the melting of co-crystals, which could be attributed to the physical crosslinking networks, which is strong enough due to the UPy units in PBS-UPy/PBF-UPy blends [37]. The intermolecular hydrogen-bonding and π-π stacking interaction mentioned above confine the chain migration and thus suppress the phase separation, leading to the formation of stable co-crystals with higher melting points compared to neat PBS-UPy. Finally, when the temperature is further raised, some recrystallized crystals of PBF-UPy would appear during such a step heating process and then totally melt at temperatures above 135 °C.

### 2.4. Crystal Structure and Morphology

**Figure 7:**
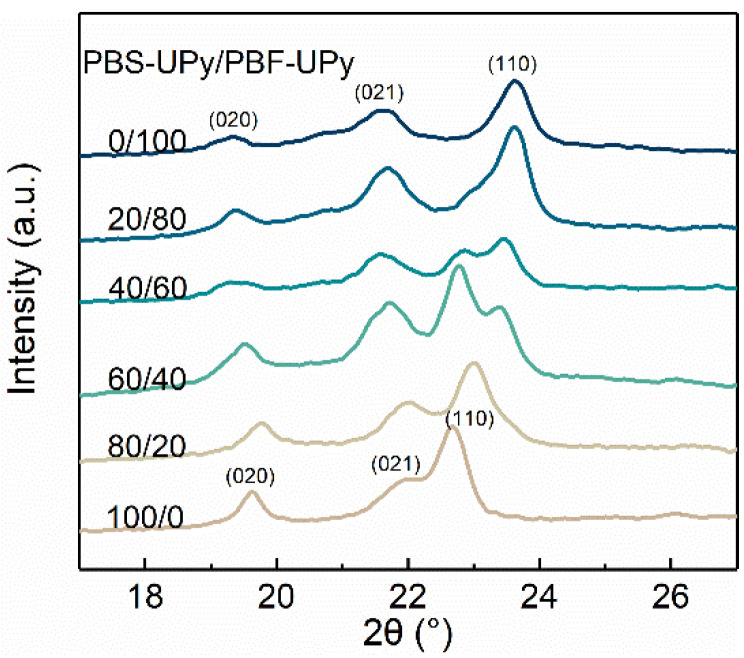
Wide angle X-ray diffractograms of PBS-UPy/PBF-UPy blends after being cooled from melt to 20 °C at a rate of 40 °C/min.

The WAXD diffractograms of the PBS-UPy/PBF-UPy blends after melt-crystallization at a rate of 40 °C/min are illustrated in Figure 7. By comparing with PBS/PBF blends [21], it is easy to find that UPy end-groups hardly change the crystal modification, but suppress the crystallization ability of the samples, resulting in broadened diffraction peaks. The (020), (021), and (110) reflection characteristics of PBS-UPy crystal are observed at 2θ = 19.6°, 21.9° and 22.7°, respectively [38]. Because of the similarity of chemical structure and packing conformation, PBF-UPy exhibits diffraction peaks located at 19.3°, 21.6°, and 23.6°, which are also, respectively, assigned to the (020), (021), and (110) interplanar spacings [21]. Instead of superposition caused by simple physical mixing, PBS-UPy/PBF-UPy-80/20 only exhibits three independent diffraction peaks ascribed to the co-crystal, which are consistent with the DSC result in Figure 4b, revealing the almost complete formation of co-crystals.

**Figure 8:**
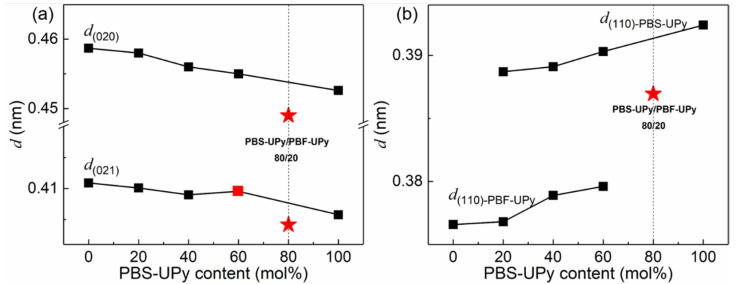
Dependences of (020), (021) interplanar spacings (**a**) and (110) interplanar spacings (**b**) on PBS-UPy content in PBS-UPy/PBF-UPy blends.

Variations of interplanar spacings (*d*) of (020), (021), and (110) with increasing PBS-UPy contents in blends are plotted in Figure 8. It can be seen that the values of d change monotonously, except for the blend of PBS-UPy/PBF-UPy-80/20. The monotonicity is similar to that of the random copolymer of poly(butylene succinate-co-butylene fumarate) (PBSF) [39]. Independent diffraction peaks and broken monotonicity are the direct evidence for the existence of only isomorphic co-crystals. Among them, the jumping values for PBS-UPy/PBF-UPy-80/20 may be attributed to some change of direction of hydrogen bonds of C–H⋯O between chain segments during the isomorphism formation [32], leading to the reduction of *d*_(020)_ and *d*_(021)_. Whereas for PBS-UPy/PBF-UPy-60/40, only the *d*_(021)_ varied discontinuously, which could be attributed to the existing mixture of co-crystals and the homo-crystals of parent components, by combining with the obvious multiple melting peaks in the above DSC thermograms.

**Figure 9:**
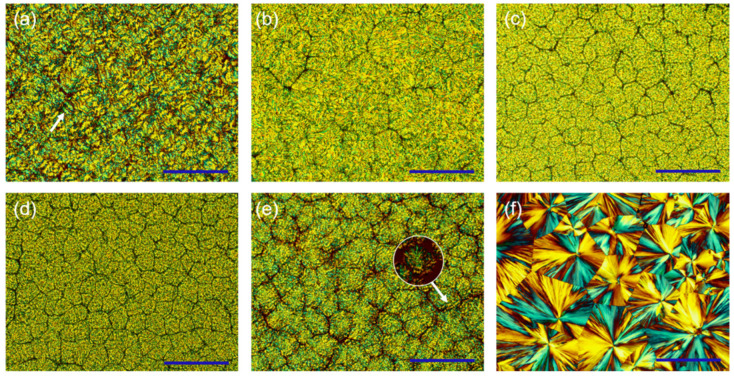
POM images of PBF-UPy (**a**) isothermally crystallized at 120 °C, PBS-UPy/PBF-Upy blends (**b**–**e**) isothermally crystallized at 115 °C and PBS-UPy (**f**) isothermally crystallized at 100 °C. (**b**–**e**) indicate PBS-UPy/PBF-UPy-20/80, PBS-UPy/PBF-UPy-40/60, PBS-UPy/PBF-UPy-60/40, PBS-UPy/PBF-UPy-80/20, respectively. All scale bars are 200 µm in length.

Since the packing of UPy units hinders the ordering of molecular chains during crystallization, the blends exhibit relatively irregular crystal morphologies. PBS-UPy/PBF-UPy blends were found to exhibit diverse spherulite morphologies under different crystallization conditions. As shown in Figure 9a, PBF-UPy exhibited irregularly banded spherulites when crystallized isothermally at 120 °C. Similarly, PBS-UPy exhibits spherulite morphology with a clear mixture of positive and negative birefringence when isothermally crystallized at 100 °C (Figure 9f). As the content of PBS-UPy increased (Figure 9b,c), the irregular ring-band disappeared and the crystals became rougher in shape. PBS-UPy/PBF-UPy-20/80 exhibited smaller spherulites compared to PBS-UPy/PBF-UPy-40/60.

For the PBS-UPy/PBF-UPy-80/20 specimen, although the spherulite morphology is still rough, it combines the characteristics of PBS-UPy and PBF-UPy spherulites. And the irregular ring-band and spherulite section with mixed positive and negative birefringence (shown by the white arrow in Figure 9e) can be vaguely distinguished. Meanwhile, PBS-UPy/PBF-UPy-80/20 can form dense crystals at a temperature higher (115 °C) than the melting point of pure PBS-UPy, indicating that the main component PBS-UPy in the blend is not crystallized alone, but co-crystallized with PBF-UPy. With the further increase in PBF-UPy content, the second component that cannot participate in the co-crystallization process will exist as an impurity, increasing the nucleation sites and making the blends PBS-UPy/PBF-UPy-60/40 (Figure 9d) exhibit smaller spherulites compared to the PBS-UPy/PBF-UPy-80/20 (Figure 9e).

## 3. Materials and Methods

### 3.1. Materials

Succinic acid (SA, AR grade), fumaric acid (FA, AR grade), 1,4-butanediol (BDO, GC grade), 1,6-hexyldiisocyanate (HDI, AR grade), 2-amino-4-hydroxy-6-methylpyrimidine (UPy, AR grade, pre-dried in a vacuum oven at 50 °C for 24 h), silica (SiO_2_, AR grade), tetrabutyl titanate (TBT, ≥99.5%), and dibutyltindilaurate (DBTD, AR grade) were purchased from Shanghai Aladdin Biochemical Technology Co., Ltd. (Shanghai, China) Chloroform (CHCl_3_, AR grade, pre-dried over molecular sieves), N,N-dimethylformamide (DMF, AR grade), petroleum ether (AR grade) and methanol (AR grade) were supplied by Beijing Chemical Reagent Factory. All reagents were used as received.

### 3.2. Fabrication of PBS-UPy/PBF-UPy Blends

Synthesis of PBS-OH and PBF-OH. Hydroxyl-terminated poly(butylene succinate) (PBS-OH) and poly(butylene fumarate) (PBF-OH) with close molecular weights were prepared by controlling the polycondensation time of the two-step melt reaction [40]. The obtained products were first dissolved in CHCl_3_ and centrifuged to remove impurities, then dried in a vacuum oven at 60 °C to obtain the pure PBS-OH and PBF-OH samples.

Synthesis of UPy-NCO. UPy (0.2 mol, thoroughly dried just before use) was added to HDI (1.2 mol) and heated to 100 °C for a sufficient reaction for 20 h. After that, excess petroleum ether was added to the above solution and the resulting white precipitate was filtered and further washed with petroleum ether. The final product, UPy-NCO, was dried in a vacuum oven at 60 °C for two days to remove any residual traces of solvent.

Synthesis of PBS-UPy and PBF-UPy. UPy-terminated PBS (PBS-UPy) and PBF (PBF-UPy) were prepared by the following procedure. UPy-NCO (8 mmol) was added into a solution of CHCl_3_ (100 mL) containing PBS-OH or PBF-OH (2 mmol), then 2 drops of DBTD were added and the mixture was continuously stirred at 60 °C for 20 h. After the sufficient reaction, 2 g of silica together with a drop of DBTD and 100 mL CHCl_3_ was added for further reaction, lasting for 1 h, to remove the excess UPy-NCO. The resulting mixture was filtered to remove impurities such as silica. The supernatant was further purified by centrifugation at 8000 rpm for 5 min and then precipitated in excess cold methanol after cooling to room temperature to obtain the final product. The obtained PBS-UPy and PBF-UPy were dried in a vacuum oven for 2 days at 60 °C.

**Figure 10:**
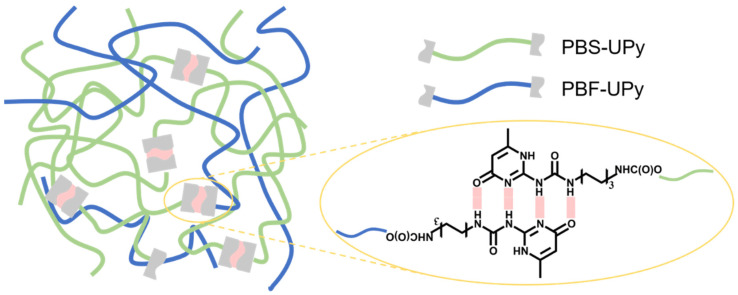
Schematic illustration of the formation of supramolecular polymer in the blends of PBS-UPy and PBF-UPy.

Fabrication of PBS-UPy/PBF-UPy blends. PBS-UPy/PBF-UPy blends were prepared by co-dissolving in DMF at 100 °C. Then, excessive cold methanol was added to precipitate the polyester blend. After sufficient precipitation, the white powder obtained by filtration was dried in a vacuum oven at 60 °C for two days. In this way, the molar ratios in blends of PBS-UPy/PBF-UPy were set as 0/100, 20/80, 40/60, 60/40, 80/20, and 100/0. The formation of supramolecular polymer in the blends between PBS-UPy and PBF-UPy entities and the self-complementary quadruple hydrogen bonds are sketched in Figure 10.

### 3.3. Characterization

Chemical structure, number-average molecular weight (*M*_n_), and the end groups of different PBS and PBF entities were characterized by ^1^H NMR spectrometer (JNM-ECA 600 MHz) using chloroform-*d* (CDCl_3_, δ = 7.2) as the solvent and tetramethylsilane (TMS, δ = 0) the standard [22].

Thermal properties of PBS/PBF and PBS-UPy/PBF-UPy blends were investigated using a differential scanning calorimeter (DSC, NETZSCH 204F1) equipped with an intercooler system under nitrogen atmosphere. Indium was used as calibration standard. All samples weighing 5–8 mg were placed in sealed aluminum pans. For the non-isothermal crystallization procedure, samples were heated to 160 °C for 3 min to erase thermal history, then cooled to 20 °C at different rates, and reheated to 160 °C at a rate of 10 °C/min. Melting temperature (*T*_m_), crystallization temperature (*T*_c_), and enthalpy (Δ*H*) were obtained from the above curves, and the values of Tm were determined in the second heating DSC curves. For the isothermal crystallization procedure, samples were first melted at 160 °C for 3 min to erase thermal history, and quickly quenched to pre-determined temperatures for complete crystallization, then they were reheated to 160 °C at a rate of 10 °C/min. The overlapping of the DSC melting endotherms was analyzed by Fit Peaks (Pro) in the Origin 2017 software with Gaussian functions.

Fourier transform infrared (FTIR) spectra were recorded using a Bruker Hyperion spectrometer equipped with a Linkam THMS600 hot stage. Spectra were recorded in transmission mode, scanning wavenumber ranged from 4000 to 1000 cm^–1^, and the final signal was averaged from 32 scans. In situ FTIR spectra were collected during the heating process after the samples were melt-crystallized on CaF_2_ wafers.

Wide angle X-ray diffraction (WAXD) measurement was carried out on a Bruker D8 Focus X-ray diffractometer using a Cu Kα radiation (λ = 0.154 nm) at room temperature. Data were collected between the 2θ range of 5.0°–40.0° with a scanning rate of 2°/min and an interval of 0.01°.

The spherulite morphology was observed using a Leica DM-2500P polarized optical microscope (POM) equipped with a Linkam THMS600 hot stage. The samples were pressed between two glass slides to obtain a thickness of around 10 µm and melted at 160 °C for 3 min to eliminate any thermal history, then quenched to the desired temperature for crystallization. A high-definition CCD digital camera was used to capture the spherulite morphology.

## 4. Conclusions

In this work, UPy-functionalized poly(butylene succinate) and poly(butylene fumarate) with close molecular weights were successfully prepared, and the crystallization and melting behavior of their blends were meticulously investigated. A novel high-level co-crystallization strategy was realized for complex supramolecular systems based on different crystalline polymers. Since complementary quadruple hydrogen-bonds act as both end-group defects and interchain linkers, a more complex co-crystallization behavior with composition and cooling rate-dependent was found. When the PBS-UPy is in the minority, a strong restriction on molecular chain migration provided by the UPy end group dominates, showing the apparently inhibited crystallization ability of the blends. As the PBS-UPy content increases, the compatibility of the two components is promoted by a quadruple hydrogen-bonding interaction, resulting in the total amount of co-crystal exceeding 98% in the PBS-UPy/PBF-UPy-80/20 blend, which provides a new approach for the preparation of a higher capability of co-crystallization in crystalline/crystalline polymer blends.

## Figures and Tables

**Table 1:** Thermal data of PBS-UPy/PBF-UPy blends during the heating process at a rate of 10 °C/min after being melt-cooled at a rate of 40 °C/min.

Samples	*T*_m1_ (°C)	Δ*H*_m1_ (J/g)	*T*_m2_ (°C)	Δ*H*_m2_ (J/g)	*T*_m3_ (°C)	Δ*H*_m3_ (J/g)
PBF-UPy	–	–	132.8	34.2	–	–
PBS-UPy/PBF-UPy-20/80	–	–	131.0	45.1	–	–
PBS-UPy/PBF-UPy-40/60	–	–	130.4	36.6	–	–
PBS-UPy/PBF-UPy-60/40	99.0	3.2	–	–	112.3	26.0
PBS-UPy/PBF-UPy-80/20	98.0	0.9	–	–	111.7	45.5
PBS-UPy	108.4/89.5	35.8	–	–	–	–

## Data Availability

Data are contained within the article.

## Supplementary Materials

The following supporting information can be downloaded at: https://www.mdpi.com/article/10.3390/molecules27207086/s1, Figure S1: DSC thermograms of PBS, PBF and PBS/PBF blends during the melt-cooling at rates of 2 °C/min and 5 °C/min, and the correspondingly subsequent heating processes at a rate of 10 °C/min.; Figure S2: The dependence of *T*_c_ and Δ*H*_c_ on the molar fraction of PBS-UPy after being melt-cooled at 10 °C/min. Figure S3: Fitting result of PBS-UPy/PBF-UPy-80/20. Figure S4: DSC thermograms of blends PBS-UPy/PBF-UPy-60/40 and PBS/PBF-80/20 during fast heating process at a rate of 40 °C/min. Figure S5: In-situ heating FTIR spectra of blend PBS-UPy/PBF-UPy-80/20 collected upon heating process from 30 to 160 °C after being melt-cooled at a rate of 40 °C/min, and changing of intensities of characteristic peaks with respect to temperature. Figure S6: In-situ heating FTIR spectra of blend PBS-UPy/PBF-UPy-80/20 collected upon heating process from 60 to 160 °C after being melt-cooled at a rate of 40 °C/min, and the trend of characteristic peak intensity as a function of temperature. Table S1: The NMR integral areas of PBS-OH, PBF-OH, PBS-UPy and PBF-UPy.
