# Supplementary material for: Promoting Co-Crystallization in Poly(butylene succinate) and Poly(butylene fumarate) Blends via End-Group Functionalization
Source: Molecules. 2022 Oct 20;27(20):7086. doi: 10.3390/molecules27207086 (PMC9608175; doi:10.3390/molecules27207086)
Supplement: Supplementary file 1 [file molecules-27-07086-s001.zip › molecules-1944191-supplementary.pdf]

Supplementary Materials

Promoting Co-crystallization in Poly(butylene succinate) and Poly(butylene fumarate) Blends via End-group Functionalization

Xue-Wei Wei[a], Cong Chen[a], Tian-Yu Wu[a,*], Li-Hai Cai[b], Hai-Mu Ye [a,*]

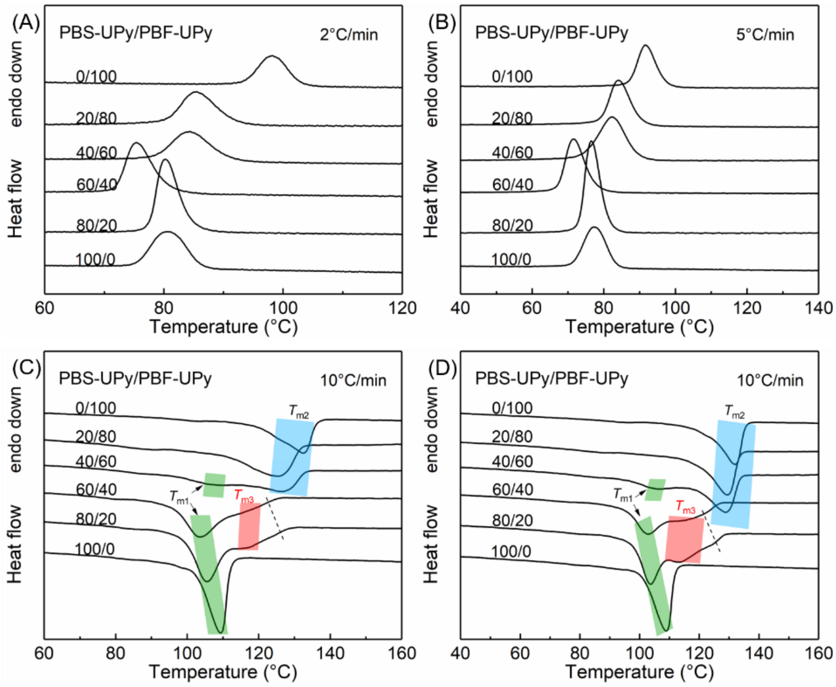

Figure S1. DSC thermograms of PBS-UPy, PBF-UPy and PBS-UPy/PBF-UPy blends during the melt-cooling at rates of 2 °C/min (A) and 5 °C/min (B), and the correspondingly subsequent heating processes at a rate of 10 °C/min (C, D).

Table S1. The NMR integral areas of PBS-OH, PBF-OH, PBS-UPy and PBF-UPy.

Samples	PBS-OH	PBF-OH	PBS-UPy	PBF-UPy
Peak1	86.5	–	44.5	–
Peak2	5.0	4.6	–	–
Peak5	–	–	1.2	0.3
Peak6	–	78.0	–	11.5

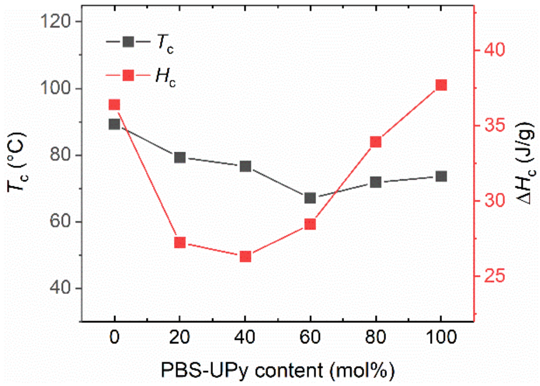

Figure S2. The dependence of T_c and ΔHc on the molar fraction of PBS-UPy after being melt-cooled at 10°C/min.

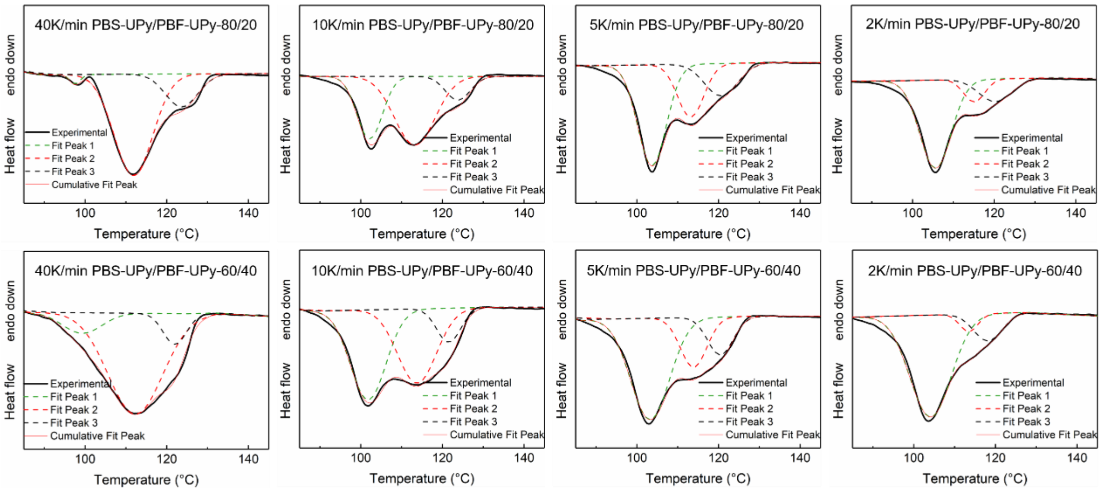

Figure S3. Fitting result of PBS-UPy/PBF-UPy blends.

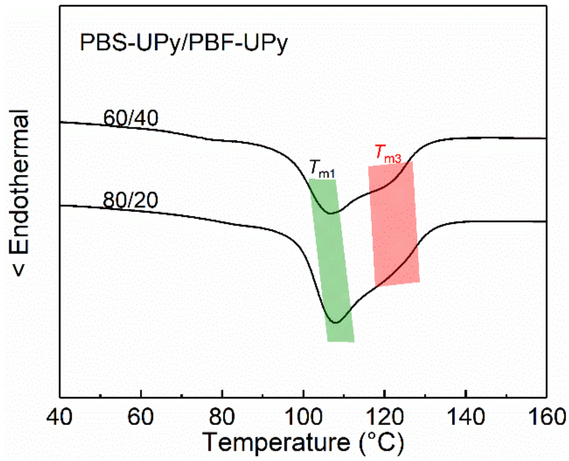

Figure S4. DSC thermograms of blends PBS-UPy/PBF-UPy-60/40 and PBS/PBF-80/20 during fast heating process at a rate of 40 °C/min.

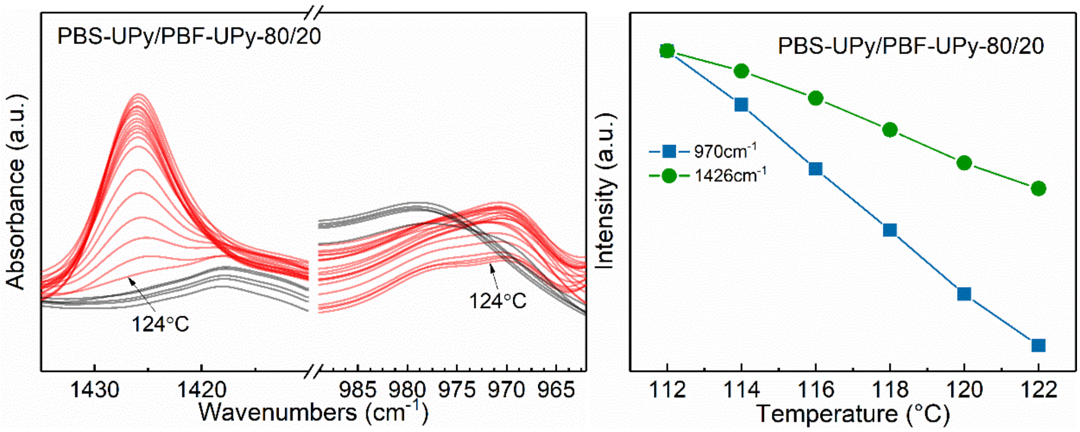

Figure S5. *In-situ* heating FTIR spectra of blend PBS-UPy/PBF-UPy-80/20 collected upon heating process from 30 to 160 °C after being melt-cooled at a rate of 40 °C/min(A), and changing of intensities of characteristic peaks with respect to temperature (B).

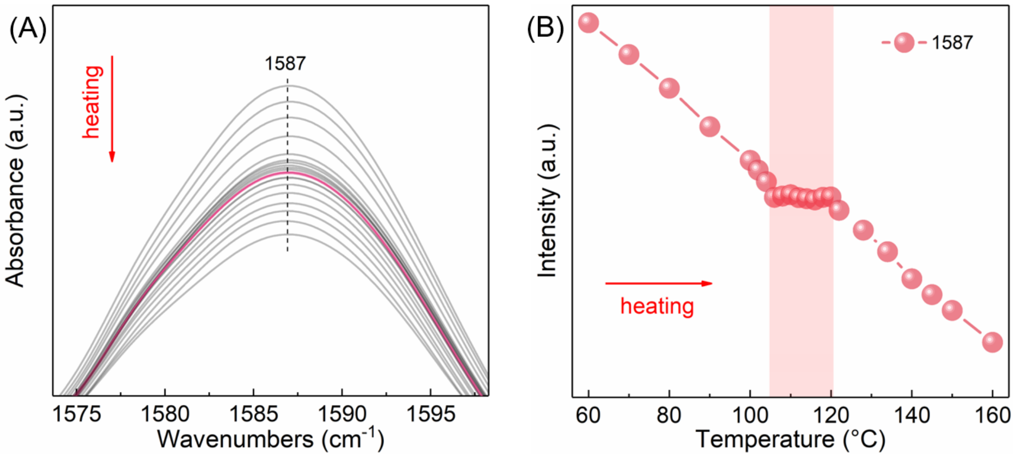

Figure S6. *In-situ* heating FTIR spectra of blend PBS-UPy/PBF-UPy-80/20 (A) collected upon heating process from 60 to 160 °C after being melt-cooled at a rate of 40 °C/min, and the trend of characteristic peak intensity as a function of temperature (B).
